# Metabolic crosstalk between oral microbiota and the host in OSCC: emerging roles of microbial metabolites in tumor initiation and progression

**DOI:** 10.3389/fcimb.2026.1778329

**Published:** 2026-02-13

**Authors:** Yajie Wu, Bo Han, Bowen Zhang, Jiyao Li, Biao Ren, Zhifei Su

**Affiliations:** 1 State Key Laboratory of Oral Diseases, National Center for Stomatology, and National Clinical Research Center for Oral Diseases, West China Hospital of Stomatology, Sichuan University, Chengdu, China; 2 Department of Endodontics, West China Hospital of Stomatology, Sichuan University, Chengdu, Sichuan, China; 3 Department of head and neck oncology, West China Hospital of Stomatology, Sichuan University, Chengdu, Sichuan, China; 4 Tianfu Jiangxi Laboratory, Chengdu, China

**Keywords:** microbial metabolites, microbiome, oral microbiota, OSCC, tumor

## Abstract

Oral squamous cell carcinoma (OSCC) is an aggressive malignancy characterized by profound metabolic reprogramming and a persistently poor clinical outcome. Beyond genetic and environmental risk factors, growing evidence indicates that dysbiosis of the oral microbiome is associated with metabolic perturbations observed in OSCC and may contribute to tumor initiation and progression. Microbiome-derived metabolites represent a previously underappreciated layer of cancer metabolism, linking microbial activity to host metabolic states, epigenetic regulation, and immune dysfunction within the tumor microenvironment. In this review, we provide a comprehensive synthesis of current evidence highlighting how microbial metabolites shape metabolic vulnerabilities in OSCC through the microbiome-metabolite-host axis. We focus on key metabolite classes, including short-chain fatty acids, tryptophan-derived metabolites, sulfur-containing compounds, and other emerging metabolic intermediates, and discuss their roles in modulating cellular energy metabolism, epigenetic remodeling, oxidative stress responses, and immune evasion. Particular emphasis is placed on the context-dependent and often dualistic functions of metabolites such as butyrate, which can exert tumor-suppressive or tumor-promoting effects depending on microbial source, concentration, and local inflammatory conditions. By integrating insights from metabolomics, microbial functional profiling, and mechanistic studies, this review underscores microbial metabolism as an integral component of OSCC pathobiology. Recognizing microbial metabolites as active metabolic regulators rather than passive byproducts provides a conceptual framework for identifying novel biomarkers and metabolic intervention strategies in OSCC.

## 1 Introduction

Cancer remains one of the most formidable challenges in modern medicine, and among its myriad forms, oral squamous cell carcinoma (OSCC) stands out as a particularly aggressive and debilitating malignancy (Zhen et al., 2025). As the predominant form of oral cancer, OSCC accounts for over 90% of cases in the oral cavity and poses a significant global oncologic burden (Jia and Wang, 2025; Zhang et al., 2025). Its aggressiveness is underscored by a high propensity for local invasion, frequent lymph node metastasis, and a persistently dismal five-year survival rate that has remained largely unchanged for decades (Asquino et al., 2025; Zhu et al., 2025). This poor prognosis is a direct consequence of late-stage diagnosis, the development of resistance to conventional therapies like radiation and chemotherapy, and a high rate of recurrence and second primary tumors (Hsu et al., 2013; Baroudi et al., 2025). While the carcinogenic roles of tobacco, alcohol, and human papillomavirus (HPV) are well-documented in oncology, they provide an incomplete explanation for the complex molecular pathogenesis of OSCC, pointing to the involvement of other, less-defined oncogenic drivers (Dorobisz et al., 2025; Kaffashian et al., 2025; Rapalska et al., 2025).

Growing evidence indicates that the tumor microenvironment (TME) plays a decisive role in OSCC progression. Within the oral TME, the resident microbiome has emerged as an integral biological component (Abbas and Javaid, 2025; Santacroce et al., 2025; Xie et al., 2025). The oral cavity harbors a complex and diverse microbial ecosystem (Sedghi et al., 2021). In a state of homeostasis, this community coexists peacefully with the host (Agrawal et al., 2025). However, a shift to dysbiosis, a microbial imbalance, is increasingly implicated in the initiation and progression of OSCC, positioning the oral microbiome as a significant factor in the field of cancer biology (Kashima et al., 2025; Liu et al., 2025). Crucially, the link between specific microbes and OSCC is not merely correlative but functionally consequential. Oncologic research has identified a distinct “inflammatory bacteriome” within OSCC tumors, enriched with pathobionts like *Fusobacterium nucleatum* (*F. nucleatum*) and *Pseudomonas aeruginosa* (*P. aeruginosa*) (Al-Hebshi et al., 2017). Emerging research elucidates that microbial metabolites act as fundamental drivers of the molecular mechanisms initiating tumorigenesis.

While studies have established that the tumor microbiome is characteristically dysbiotic, its influence on oncogenesis is multifaceted, with metabolite production standing out as a central mechanism (Duan et al., 2025). Microbial metabolites are regarded as a central effector molecule in OSCC tumor biology (Lou et al., 2025). They can infiltrate host cells, act as signaling molecules, reprogram host cell metabolism, induce genomic instability, and sculpt an immunosuppressive niche, thereby directly promoting the hallmarks of cancer. Therefore, the imperative to synthesize this field is paramount. Metabolomic analyses have been pivotal in uncovering this functional link: profiling of saliva and tissue from OSCC patients, as exemplified by the work of Ohshima et al., has identified specific onco-metabolites, such as elevated choline, branched-chain amino acids, and altered kynurenine pathway products, that are actively produced by the resident microbiota and contribute to tumor progression (Ohshima et al., 2017).

Furthermore, the concept of the dual effects of microbial metabolites holds profound significance for precise cancer treatment (Zhao et al., 2025a; Bishoyi et al., 2026). The metabolic activities of tumor-associated microorganisms in the tumor microenvironment are highly complex. This review will critically analyze how metabolites exert both positive and negative influences on tumor development. Understanding this duality is crucial for developing therapies that selectively target pro-tumor metabolic pathways while preserving the functions of anti-cancer microorganisms.

In addition, the microbiome-metabolite-immune axis as a fundamental mechanism of immune evasion in OSCC, a major hurdle in cancer immunotherapy. The immunosuppressive TME is a hallmark of many cancers, including OSCC (Lin et al., 2021; Zhao et al., 2025b). Regulatory T cells (Tregs) are master regulators of this immune suppression, and their function is metabolically dependent (Gan et al., 2024). Microbial metabolites are potent manipulators of this process. Microbial metabolites have been implicated in shaping this landscape. For example, bacteria-driven tryptophan metabolism toward kynurenine may promote Treg expansion via aryl hydrocarbon receptor signaling, while simultaneously limiting cytotoxic T-cell function (Kenison et al., 2021; Zhou et al., 2024). Together, these findings support a mechanistic link between microbial metabolic pathways and immune evasion, although the strength of causal inference in humans remains limited.

**Figure 1:**
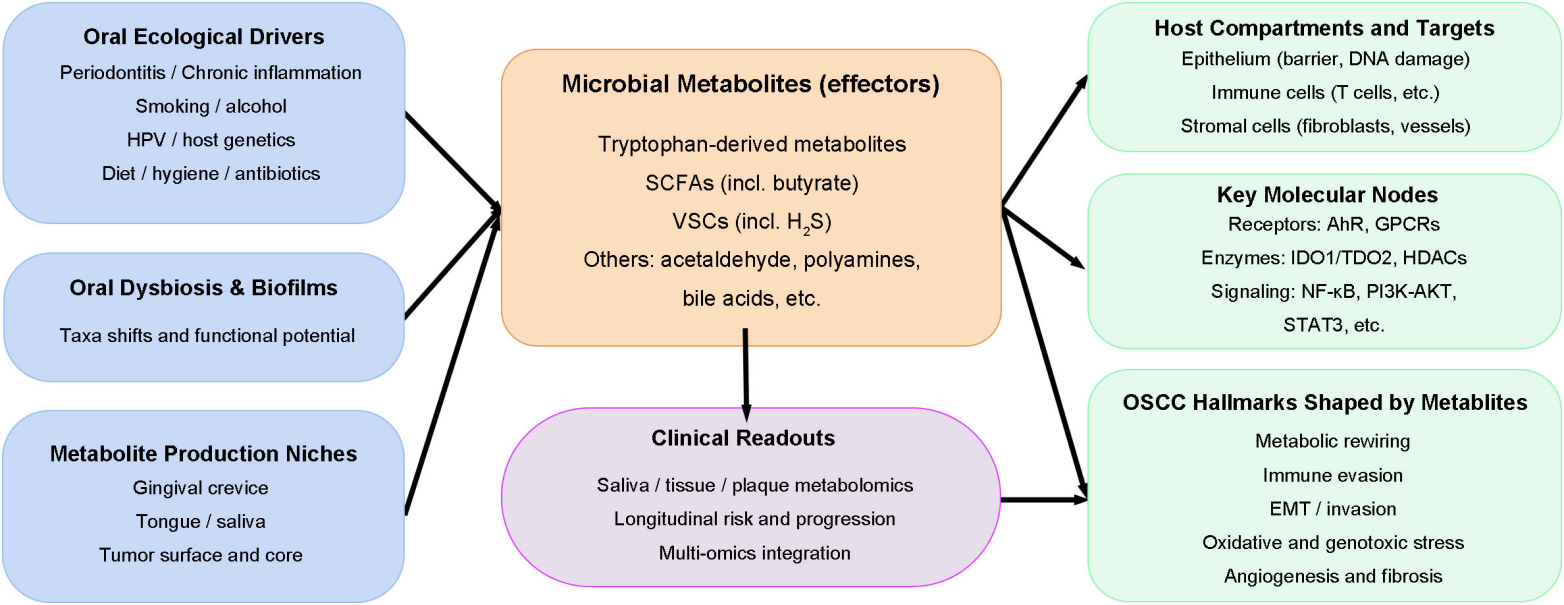
Conceptual overview of the oral microbiome-metabolite-host axis in OSCC.

In this review, we move beyond descriptive microbial taxonomic signatures and instead center on microbial metabolites as functional effectors that couple oral microbial activity to host metabolic states within the tumor microenvironment. We next summarize major metabolite classes and their mechanistic links to OSCC (**Figure 1**). Our analysis moves beyond descriptive cataloguing of microbial communities toward a mechanistic integration of the two fields. We focus on how microbial metabolites establish critical host vulnerabilities, including immunometabolic remodeling, epigenetic dysregulation, and compromises in redox homeostasis and epithelial integrity, thereby elucidating actionable insights into OSCC pathogenesis. Collectively, this review aims to firmly establish targeting the “microbiome-metabolite-cancer” axis as a groundbreaking and translational frontier in the oncologic management of OSCC.

## 1 Oral microbiota dysbiosis and OSCC

### 1.1 Differences in oral microbiota composition between OSCC patients and healthy individuals

Comparative studies have consistently revealed distinct microbial signatures in OSCC patients compared to healthy controls (Guerrero-Preston et al., 2016; Zhao et al., 2017; Lyu et al., 2025). Significant shifts in microbial composition are observed as oral mucosa transitions from healthy to precancerous and cancerous states (Lee et al., 2017). For instance, OSCC tissues exhibit a marked depletion of *Actinobacteria* and *Cyanobacteria*, along with decreased abundance of *Streptococcus*, *Haemophilus*, *Porphyromonas*, and *Actinomyces* (Guerrero-Preston et al., 2016; Wei et al., 2025). Conversely, increases in *Fusobacterium*, *Peptostreptococcus*, *Capnocytophaga*, and *Alloprevotella* are frequently reported in OSCC samples (Zhao et al., 2017; Granato et al., 2021; Lyu et al., 2023). Salivary microbiome analyses also reflect these changes, with notable increases in *Prevotella* and reductions in *Neisseria* in cancer patients (Han et al., 2025).

Longitudinal and cross-sectional studies further support these trends. For example, *Abiotrophia*, *Acinetobacter*, *Peptostreptococcus*, and *Staphylococcus* remain elevated in OSCC patients even after surgical intervention (Soghli et al., 2025). Community-level analyses reveal consistent microbial alterations in the TME, including increased *Fusobacteria* and *Proteobacteria*, and decreased *Firmicutes* and *Actinobacteria*, with more pronounced changes in metastatic samples (Eun et al., 2021).

### 1.2 Key carcinogenic bacteria in oral cancer

Several bacterial species have been implicated in oral carcinogenesis, including both “cross-domain” pathogens found in multiple body sites and oral-specific taxa. *F. nucleatum*, a well-studied oral pathobiont, is also implicated in colorectal cancer (Gao et al., 2025). It promotes OSCC progression through mechanisms such as epithelial-mesenchymal transition (EMT) via the lncRNA MIR4435−2HG/miR−296−5p/Akt2/SNAI1 pathway and activation of STAT3 (Munoz-Grez et al., 2025). In parallel, *Porphyromonas gingivalis* (*P. gingivalis*), *Treponema denticola*, and *Prevotella intermedia* are also strongly associated with periodontitis and OSCC. *P. gingivalis* activates multiple signaling pathways (e.g., ERK1/2-Ets1, p38/HSP27, PAR2/NF−κB) that enhance matrix metalloproteinase expression and tumor invasiveness (Urzica et al., 2025). Both *F. nucleatum* and *P. gingivalis* activate the TLR/MyD88-triggered integrin/FAK pathway, further promoting tumor progression (Chen et al., 2018; Liu et al., 2020). In addition, *Streptococcus pneumoniae* and other *Streptococci* are also recognized for their pro-inflammatory roles and potential involvement in both oral and systemic carcinogenesis (Su et al., 2021).

### 1.3 From dysbiosis to altered metabolite profiles: a logical progression

Oral dysbiosis contributes to carcinogenesis not only through direct microbial interactions but also via metabolic reprogramming. Shifts in microbial composition, such as increases in *Streptococcus*, *Abiotrophia*, and *Leuconostoc*, and decreases in *Prevotella*, *Haemophilus*, and *Neisseria*, alter the production of short-chain fatty acids (SCFAs), key anti-inflammatory metabolites (Perera et al., 2017). Reduced SCFA levels and downregulation of free fatty acid receptor 2 (FFAR2) expression disrupt immune homeostasis and promote a pro-inflammatory state (Magrin et al., 2020).

Dysbiosis also activates pro-inflammatory pathways such as TNFAIP8 and IL−6/STAT3, which are known to support chronic inflammation and tumor development (Nakkarach et al., 2021). Furthermore, microbial dysbiosis influences the production of other metabolites, including lipoteichoic acid from *Streptococci*, which perpetuates inflammation and exacerbates mucosal barrier dysfunction (Yau et al., 2018). This metabolic dysregulation, coupled with immune evasion and persistent inflammation, creates a microenvironment conducive to tumor initiation and progression.

## 2 Mechanistic insights into key microbial metabolites in OSCC

**Figure 2:**
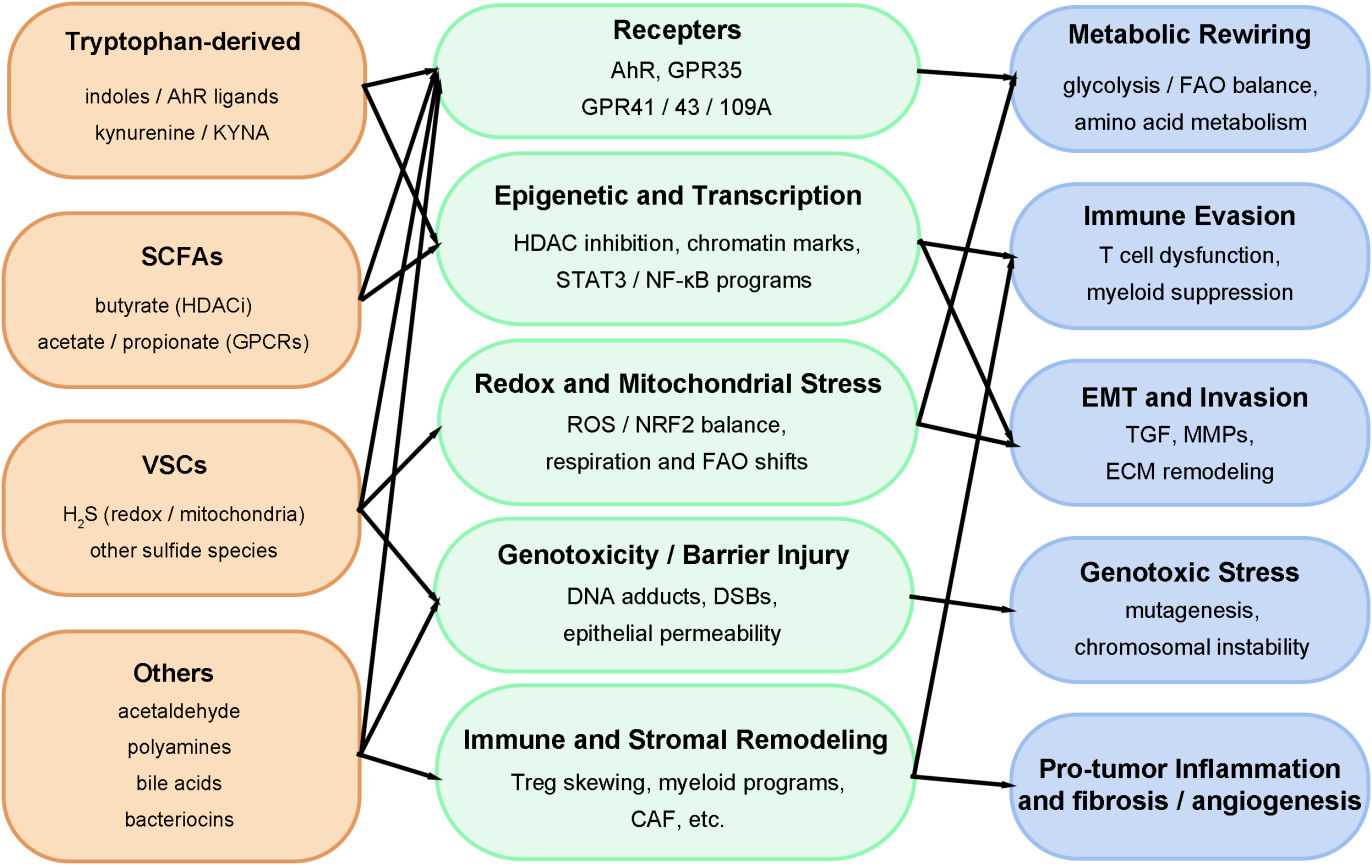
Pathway map linking representative microbial metabolites to host nodes and phenotypes in OSCC.

This section is organized by major classes of microbial metabolites implicated in OSCC pathogenesis: tryptophan-derived metabolites (Section 2.1), SCFAs including butyrate (Section 2.2), volatile sulfur compounds (VSCs) such as hydrogen sulfide (H_2_S) (Section 2.3), and other relevant metabolites (Section 2.4)-including acetaldehyde, polyamines, bile acids, and bacteriocins. While their microbial sources and physiological concentrations vary across oral niches and sample types, these metabolites collectively converge on a core set of host cellular programs: metabolic rewiring, epigenetic remodeling, redox and mitochondrial stress, barrier disruption and genotoxic damage, and immune-stromal reprogramming. Together, these pathways critically shape the phenotypic evolution of OSCC. To visually synthesize these interactions, **Figure 2** presents an integrated pathway map, and **Table 1** provides a systematic summary of key metabolites, their microbial producers, host targets, and associated evidence, serving as a navigational guide for the detailed discussions in Sections 2.1 through 2.4.

**Table 1:** Key microbial metabolites in OSCC (summary).

Class	Key metabolites	Representative producers	Primary host targets	Main mechanisms (summary)	OSCC relevance	Common sample type
Tryptophan-derived	Indoles (AhR ligands)	Fusobacterium, Prevotella, Porphyromonas (context- dependent)	AhR	Immune tuning; epithelial differentiation; barrier programs	Biomarker candidate; mechanistic links emerging	Saliva / tissue
	Kynurenine / KYNA	Community/host enzyme axis (IDO1/TDO2; microbial contribution debated)	IDO1/TDO2; GPR35 (KYNA)	Immune suppression; metabolic signaling; pro- tumor inflammation	Biomarker + therapeutic enzyme axis	Tissue / saliva
SCFAs	Butyrate	Anaerobic periodontal consortia; Veillonella/Prevotella (varies)	HDACs; GPR41/43/109A	Epigenetic remodeling; dose-dependent anti/pro- tumor effects; immune modulation	Mechanistically developed; context-dependent	GCF / saliva / plaque
	Acetate/Propionate	Mixed oral anaerobes	GPR41/43	Immunometabolic effects; barrier and cytokine programs	Associations reported; mechanisms less resolved in OSCC	Saliva / plaque
VSCs	Hydrogen sulfide (H_2_S)	Fusobacterium, Porphyromonas, Treponema (varies)	Mitochondria/redox sensors (indirect)	Redox stress; mitochondrial dysfunction; DNA damage; inflammation	Emerging driver + risk marker	Saliva / tissue
	Acetaldehyde	Alcohol-metabolizing oral microbes; Candida also relevant	DNA (adduct formation)	Genotoxicity; mutagenesis; barrier injury	High translational relevance (risk modifier)	Saliva / mucosa
	Polyamines (putrescine, spermidine)	Diverse oral microbes; biofilm metabolism	Polyamine transport/metabolic enzymes	Proliferation support; stress tolerance; immune effects	Emerging; needs OSCC- specific causal studies	Saliva / tissue
Others	Bile acids (secondary forms)	Microbial conversion possible when bile acids enter oral cavity	FXR/TGR5 (conceptual)	Epithelial stress & inflammation (hypothesized)	Emerging; reflux-linked exposure a key context	Saliva
	Bacteriocins / peptides	Streptococcus and others	Membranes/immune recognition (varies)	Community shaping; indirect tumor effects via ecology	Indirect; ecology-focused	Plaque / saliva

### 2.1 Tryptophan metabolites

The role of microbial metabolites in the progression of OSCC is increasingly evident. A central mechanism in OSCC pathogenesis and immune evasion involves the metabolism of tryptophan through the kynurenine pathway (Lou et al., 2025). In the TME, tryptophan can be converted to kynurenine and downstream metabolites such as kynurenic acid (KYNA), which have been reported to be elevated in OSCC tissues and patient saliva compared with healthy controls, indicating dysregulated tryptophan metabolism in OSCC (Walczak et al., 2020; Zhou et al., 2024).

This metabolic alteration is further driven by specific oral microorganisms, including *Streptococcus mutans* (*S. mutans*), which colonizes OSCC tissues and enhances KYNA production via its surface protein antigen C (PAC). The accumulation of KYNA not only disrupts local immune responses but also promotes the expansion of regulatory T cells (Tregs), thereby reinforcing an immunosuppressive TME (Zhou et al., 2024).

Microbial dysbiosis also contributes critically to OSCC progression. The bacteriome in OSCC patients is enriched with pro-inflammatory species such as *F. nucleatum* and *P. aeruginosa*, which can influence metabolic pathways including the kynurenine axis (Li et al., 2024, 2025). This promotes an inflammatory microenvironment that exacerbates tissue damage, epithelial proliferation, and tumor development. The altered oral microbiome further facilitates metabolic reprogramming by stimulating the secretion of inflammatory cytokines and metabolites involved in tryptophan degradation, thereby accelerating OSCC progression. At the host level, Tregs in OSCC display pronounced metabolic plasticity, relying on coordinated regulation of glycolysis, fatty acid oxidation, and amino acid metabolism to maintain suppressive function. Activation of the kynurenine–aryl hydrocarbon receptor (AhR) axis has been reported to enhance Treg stability and suppress effector T-cell responses, providing a mechanistic framework by which altered tryptophan metabolism may facilitate immune evasion in OSCC (Kenison et al., 2021; Xu et al., 2023).

In summary, microbial dysbiosis in OSCC not only alters microbial composition but also directly modulates host metabolic pathways, particularly tryptophan metabolism. This interaction between the oral microbiome and host metabolism promotes immune suppression and facilitates tumor immune evasion. From a diagnostic perspective, elevated kynurenine and KYNA levels consistently observed in OSCC tissues and patient saliva indicate that dysregulated tryptophan metabolism may serve as a potential metabolic biomarker of disease presence and immune status. In contrast, therapeutic strategies targeting the kynurenine-AHR axis, including enzyme inhibition or microbiome modulation, remain largely preclinical and are primarily supported by mechanistic and animal studies.

### 2.2 SCFAs (including butyrate)

SCFAs such as acetate and propionate are produced by oral microbiota through the fermentation of dietary fibers and resistant starches. SCFAs, in particular, are involved in modulating immune responses in the oral cavity (Baima et al., 2025). These metabolites can influence the balance between pro-inflammatory and anti-inflammatory cytokines. In the context of OSCC, SCFAs can either promote or inhibit cancer progression depending on their concentration and the microbial community’s composition. This interplay suggests that microbial dysbiosis and the subsequent metabolic disturbances contribute to OSCC progression by affecting immune cell infiltration and inflammatory pathways (Nouri et al., 2023).

Among SCFAs, butyrate has received particular attention due to its diverse biological activities (Zang et al., 2022). Notably, physiological butyrate levels in the oral cavity (e.g., saliva/gingival crevicular fluid) are typically far lower than those in the colonic lumen, and this exposure gap should be considered when interpreting dose-dependent dual effects and extrapolating findings across tissues. In many cancer types, including colorectal and lung cancer, butyrate has been classically regarded as a tumor-suppressive metabolite through mechanisms such as HDAC inhibition, activation of G-protein-coupled receptors, and modulation of epithelial and immune cell function (Goncalves and Martel, 2013; Bishoyi et al., 2026). However, emerging evidence in OSCC suggests a more nuanced, context-dependent role in which butyrate can also participate in pro-tumorigenic processes.

The relationship between periodontitis and OSCC is well-documented, with periodontal pathogens such as *F. nucleatum* and *P. gingivalis* found to be enriched in OSCC tissues compared to normal tissue (Plottel and Blaser, 2011; Li et al., 2018). Notably, *F. nucleatum*, a bacterium commonly associated with periodontitis, has been shown to produce butyrate by metabolizing L-glutamate released by OSCC cells. Mechanistically, *F. nucleatum* upregulates the cystine/glutamate antiporter SLC7A11 in OSCC cells, promoting glutamate efflux and providing a continuous carbon source that can be catabolized into butyrate (Munoz-Grez et al., 2025). This microbial activity not only provides energy to the bacteria but also reshapes the metabolic landscape of the tumor niche, supplying a metabolite that can influence cancer cell growth, stress responses, and interaction with surrounding stromal and immune cells.

Butyrate’s effects on cancer cells are strongly dose and context dependent. Studies in OSCC models have shown that at lower or intermediate concentrations (for example, in the 100–1000 nmol/L range), butyrate can stimulate tumorsphere growth, enhance cellular proliferation, and promote features associated with EMT, including increased migration and invasion (Tsai et al., 2020). Consistent with these findings, butyrate exposure has been reported to upregulate EMT-related markers such as SNAI1, Vimentin, and N-cadherin, while downregulating epithelial markers, thereby facilitating a more invasive phenotype (Du et al., 2014; Zang et al., 2022). In this setting, butyrate may act in concert with other bacterial products (e.g., LPS, proteases) and chronic inflammatory signals to drive matrix remodeling, disrupt cell-cell adhesion, and support local dissemination of OSCC cells. These data support a model in which butyrate derived from dysbiotic periodontal communities can act as a pro-tumorigenic mediator when present at sub-cytotoxic levels in a chronically inflamed microenvironment.

However, the role of butyrate in OSCC progression is not entirely straightforward. While it displays tumor-promoting effects at certain concentrations and in microbiome-driven contexts, its overall impact on cancer progression appears to depend on the balance between these pro-tumoral actions and its intrinsic capacity to function as an HDAC inhibitor and immunomodulatory metabolite (Yue et al., 2022). A substantial body of work indicates that, at higher concentrations or when delivered as a pharmacologic agent, butyrate can inhibit OSCC cell proliferation, induce cell-cycle arrest, and reduce invasive behavior (Zang et al., 2022). Sodium butyrate (NaB), a stable and widely used butyrate derivative, has been shown to suppress OSCC cell growth and invasion by downregulating HDAC1 (Gillenwater et al., 2000). This, in turn, relieves epigenetic repression of the tumor-suppressor gene HSPB7, leading to its upregulation and consequent restriction of OSCC cell survival and motility (Yue et al., 2022). Other studies report that butyrate can decrease the expression of adhesion molecules such as ICAM-1 and modulate inflammatory enzymes like COX-2 and secretory phospholipase A2-X in oral cancer cells, further supporting its potential anti-tumor effects (Gharat et al., 2016).

Furthermore, insights from broader SCFA research suggest that butyrate may also influence OSCC through additional layers of regulation, including effects on angiogenesis, ferroptosis sensitivity, and anti-tumor immunity (Zhong et al., 2025). By inhibiting HDACs and engaging GPCR signaling, butyrate can alter chromatin accessibility, regulate VEGF and its co-receptors, and modulate the activity of T cells, dendritic cells, and NK cells (Bishoyi et al., 2026). Although these mechanisms have been characterized more extensively in other malignancies, they provide a conceptual framework for understanding how butyrate might shape the OSCC TME beyond direct actions on tumor cells, for instance by influencing immune checkpoint signaling or the balance between effector and regulatory immune subsets.

In summary, butyrate, as a microbial metabolite, plays a complex and dual role in OSCC progression. Its presence in the oral cavity, primarily produced by periodontal and other anaerobic bacteria, can contribute to tumor growth and invasion through mechanisms such as EMT induction, metabolic support of pathogenic species like *F. nucleatum*, and reinforcement of chronic inflammation. At the same time, butyrate retains the potential to exert tumor-suppressive effects via HDAC inhibition, epigenetic reprogramming, and immune modulation, particularly when present at higher concentrations or delivered exogenously. The net effect of butyrate in OSCC thus depends on its local concentration, source (commensal versus pathogenic microbiota), and interaction with host signaling pathways. This duality not only highlights butyrate as a candidate biomarker reflecting dysbiotic metabolic activity in OSCC, but also positions the butyrate axis as a promising, yet challenging, therapeutic target that will require careful tuning of microbiome and metabolite levels to selectively harness its anti-tumor potential.

### 2.3 VSCs (including hydrogen sulfide)

VSCs, such as methyl mercaptan, are another group of metabolites produced by oral bacteria like *P. gingivalis* and *F. nucleatum* (Kwon et al., 2022). These compounds have been implicated in the development of OSCC by promoting an inflammatory microenvironment. VSCs can activate pro-inflammatory cytokine production, leading to immune system dysregulation and chronic inflammation (Krespi et al., 2006). This chronic inflammatory state is a known risk factor for the initiation and progression of OSCC. In addition, VSCs may disrupt epithelial cell barriers, promoting the infiltration of carcinogenic bacteria and enhancing tumor cell proliferation, invasion, and metastasis.

Hydrogen sulfide (H_2_S), a VSC, is produced both endogenously and by oral bacteria, playing a significant role in the pathogenesis of oral diseases, including OSCC (Wu et al., 2022). In OSCC, the concentration of H_2_S is markedly elevated compared to adjacent benign mucosal tissue, suggesting its involvement in the malignant transformation of oral cells. The enzymes responsible for H_2_S synthesis, cystathionine β-synthase (CBS), cystathionine γ-lyase (CSE), and 3-mercaptopyruvate sulfur transferase (3-MST), are upregulated in OSCC tissues, contributing to the increased H_2_S levels observed in these malignancies (Meram et al., 2018). This elevation in H_2_S concentration correlates with the activation of key oncogenic pathways, such as the MAPK and STAT3 signaling cascades, which are implicated in tumor growth, survival, and metastasis. The presence of H_2_S in the OSCC microenvironment also influences the oxidative stress balance, promoting DNA damage and facilitating a pro-inflammatory state conducive to tumor progression (Vitvitsky et al., 2012; Ma et al., 2015; Wu et al., 2022).

Moreover, H_2_S-producing bacteria, which thrive in the oral cavity, exacerbate the effects of this gas by increasing its local concentration, further supporting the inflammatory and carcinogenic processes in OSCC (Kwon et al., 2022). The correlation between elevated levels of H_2_S and OSCC progression also underscores its potential as a non-invasive diagnostic marker. Studies have shown that exhaled breath analysis, which detects H_2_S, could serve as a promising diagnostic tool for OSCC.

In conclusion, H_2_S is not only a key microbial metabolite in oral health but also a crucial factor in the pathogenesis of OSCC. Its dual role in promoting oxidative stress, inflammation, and cell survival makes it a potential target for therapeutic intervention in OSCC, as well as a valuable biomarker for early detection of the disease.

### 2.4 others

Various microbial metabolites produced by oral bacteria also have been shown to influence the inflammatory microenvironment, immune response, and cellular signaling pathways that contribute to cancer progression.

Bacteriocins, antimicrobial peptides produced by beneficial bacteria in the oral microbiota, have also been studied for their potential role in OSCC (Kamarajan et al., 2020). These molecules, produced by species such as *Lactobacillus* and *Streptococcus*, have antimicrobial properties that inhibit the growth of pathogenic bacteria like *P. gingivalis* and *F. nucleatum*. Since these pathogens are closely associated with OSCC development, bacteriocins might help reduce the pathogenic microbial load, thereby maintaining a healthier microbiome. Some bacteriocins also possess anti-inflammatory properties that may prevent the inflammatory signaling cascades typically observed in OSCC, further suggesting their potential as therapeutic agents.

Secondary bile acids are microbially generated derivatives of primary bile acids; importantly, primary/total bile acids are not produced in the oral cavity but can be detected in saliva mainly due to gastroduodenal reflux (GERD/LPR), which delivers bile-containing refluxate into the upper aerodigestive tract (Krause et al., 2024; Thakur et al., 2025). Accordingly, any discussion of bile acid-oral epithelium interactions should be interpreted as exposure in reflux-associated contexts, and OSCC-relevant evidence remains emerging and largely indirect rather than OSCC-specific causal proof (De Corso et al., 2021). These bile acids can affect various signaling pathways that are dysregulated in cancer, such as the Wnt/β-catenin and JAK-STAT pathways (Akhtar et al., 2025). In particular, certain secondary bile acids have been linked to increased carcinogenesis by promoting DNA damage and mutations in oral epithelial cells. The role of these metabolites in OSCC depends heavily on the balance between beneficial and pathogenic microbes in the oral cavity (Abusleme et al., 2013). Dysbiosis, marked by an overabundance of certain bacteria that produce harmful secondary bile acids, could increase the risk of cancer development.

Beyond established microbial metabolites, lactate is emerging as a key player that actively remodels the tumor microenvironment, representing a potentially overlooked node within the oral “microbiome-metabolite-host” axis (Gong et al., 2026). In cancer, heightened aerobic glycolysis (the Warburg effect) leads to lactate accumulation within tumors (Hanahan and Weinberg, 2011). Rather than a mere metabolic waste product, lactate is now recognized as a pleiotropic signaling molecule implicated in disease progression. It serves as both a readout of cellular metabolic activity and a driver that reinforces glycolytic flux through feedforward metabolic-transcriptional coupling. Mechanistically, lactate shuttling between cells-mediated by monocarboxylate transporters (MCT1-MCT4)-allows it to function as an energy substrate and, importantly, as a signaling messenger that coordinates metabolic crosstalk in the tumor niche (Singh et al., 2023). Notably, lactate-induced protein and histone lactylation provides a direct link between metabolism and epigenetic regulation, contributing to immunosuppressive reprogramming, such as promoting M2-like macrophage polarization and recruiting tumor-associated macrophages via the STAT3-CCL2 axis (Sun et al., 2025). In head and neck squamous cell carcinoma, elevated histone lactylation (e.g., H3K9la) has been associated with poorer responses to immunotherapy, highlighting its potential translational relevance in OSCC (Gong et al., 2026). Collectively, these insights argue for incorporating lactate and lactylation into the metabolic landscape of OSCC. Future studies should clarify the relative contributions of host- versus microbiota-derived lactate pools and define context-dependent thresholds that dictate whether lactate exerts pro-tumor or immunomodulatory effects.

Acetaldehyde (ACH) serves as a prototypical microbial, chemical effector that bridges conventional risk factors such as alcohol and smoking to local carcinogenic metabolism in the oral cavity. Ethanol is oxidized to acetaldehyde by oral microorganisms via microbial alcohol dehydrogenase activity, with a wide range of taxa, including oral streptococci, *Neisseria* spp., and *Candida*, capable of generating substantial acetaldehyde under physiologically relevant ethanol exposure (Marttila et al., 2013; Naz and Zafar, 2025). Culture-based studies using site-specific isolates from OSCC lesions, oral lichenoid lesions, and healthy mucosa have demonstrated that many of these microbes frequently produce mutagenic concentrations of acetaldehyde, with elevated levels observed particularly in smokers (Marttila et al., 2013). This supports a model of microbial ecological selection under chronic exposure. Mechanistically, acetaldehyde acts as a direct chemical carcinogen by forming DNA adducts and crosslinks, impairing DNA synthesis and repair. In OSCC tissues, microbial acetaldehyde production correlates with suprabasal p53 immunostaining, aligning with documented *TP53* mutagenesis in adjacent epithelia driven by acetaldehyde exposure (Marttila et al., 2021). Notably, oral leukoplakia is a key oral potentially malignant disorder that exhibits enrichment of taxa previously linked to acetaldehyde generation (e.g., *Rothia mucilaginosa*, *Streptococcus parasanguinis*, *Streptococcus salivarius*), further supporting the role of acetaldehyde as a functional metabolic axis active early in malignant transformation (Ramos et al., 2020; Galvin et al., 2025).

Polyamines, such as putrescine, spermidine, and spermine, are small cationic metabolites that can be synthesized within oral biofilms and often accumulate in tumors. In bacteria, putrescine is typically generated from arginine or ornithine through decarboxylation pathways. For example, *S. mutans* employs the agmatine deiminase system to convert agmatine (derived from arginine) into putrescine. Additionally, *F. nucleatum* has been shown to stimulate putrescine production, indicating a likely microbial origin of polyamines in dysbiotic oral environments (Zhi et al., 2024). In OSCC, polyamine metabolism is commonly upregulated: malignant epithelial regions exhibit elevated levels of spermidine or spermine, along with heightened activity of key biosynthetic enzymes (ODC1-SRM-SMS), in line with the established roles of polyamines in supporting cellular proliferation and remodeling the tumor microenvironment (Tang et al., 2023).

Taken together, these microbial metabolites, SCFAs, VSCs, bacteriocins, and secondary bile acids, etc., illustrate the complex relationship between the oral microbiota and OSCC. Dysbiosis, or an imbalance in microbial populations, can lead to a shift in metabolite production that promotes carcinogenesis through inflammation, immune modulation, and cellular damage. Further research into these metabolites may provide novel therapeutic approaches for managing OSCC by targeting microbial pathways or manipulating the oral microbiome to restore a healthy balance.

## 3 The dual role of microbial metabolites: biomarkers and therapeutic targets in OSCC

Early detection and effective treatment remain challenging, largely due to the lack of reliable biomarkers for early diagnosis and the limited therapeutic options available. Emerging evidence suggests that microbial metabolites, produced by the diverse microbiota in the oral cavity, play a significant role in the pathogenesis of OSCC. These metabolites not only contribute to disease progression but also offer promising opportunities for novel diagnostic and therapeutic strategies (Soghli et al., 2025). By profiling specific microbial metabolites in the oral cavity, we can gain valuable insights into disease mechanisms and identify potential biomarkers for early detection. Moreover, targeting the metabolic pathways of these microbial products presents an innovative approach to treatment, offering a means to halt or slow down OSCC progression. This section will explore the potential of microbial metabolites as both diagnostic biomarkers and therapeutic targets in OSCC, focusing on their role in disease detection, risk prediction, and treatment intervention.

### 3.1 Diagnostic biomarkers

The identification of microbial metabolites in saliva or tissue metabolic profiles has emerged as a promising strategy for the early diagnosis of OSCC and the prediction of malignant transformation risk. Specific metabolites and their ratios in the oral environment can serve as potential biomarkers for OSCC detection. For instance, the elevated levels of KYNA and the ratio of certain SCFAs, such as acetate, propionate, and butyrate, have been linked to OSCC development (Zhou et al., 2024). The balance between beneficial and pathogenic microbiota is reflected in these metabolic signatures, providing insights into cancer progression (Duan et al., 2025). Furthermore, H_2_S, a metabolite produced by oral bacteria, has shown potential as a biomarker for OSCC, with altered levels correlating to cancerous changes in the oral mucosa. These metabolic profiles can be utilized in non-invasive diagnostics, such as saliva-based tests, to detect OSCC at early stages or predict the risk of malignant transformation in premalignant lesions (Wu et al., 2022).

### 3.2 Therapeutic targets

Microbial metabolites not only hold potential as biomarkers but also present novel therapeutic targets for OSCC treatment. The targeting of specific metabolic enzymes and receptors could offer a way to halt or slow OSCC progression.

#### 3.2.1 Targeting metabolic enzymes

Inhibiting the enzymes involved in the production of oncogenic metabolites is a promising strategy. For example, targeting indoleamine 2,3-dioxygenase 1 (IDO1) and tryptophan 2,3-dioxygenase 2 (TDO2), enzymes responsible for the catabolism of tryptophan into KYNA, could prevent the accumulation of this carcinogenic metabolite (Abd El-Fattah, 2022). Developing specific inhibitors for these enzymes could block the synthesis of KYNA, which is known to promote OSCC cell proliferation and immune escape, thus providing a therapeutic approach (Zhou et al., 2024).

#### 3.2.2 Targeting metabolite receptors

Another potential strategy is targeting receptors involved in the signaling pathways of microbial metabolites. For instance, G protein-coupled receptor 35 (GPR35), which is activated by certain microbial metabolites like KYNA, plays a role in regulating immune responses and inflammation. By targeting GPR35, it may be possible to modulate the immune microenvironment in OSCC, improving the efficacy of existing treatments or reducing tumor growth (Walczak et al., 2020).

#### 3.2.3 Microbiome intervention

Modulating the oral microbiota offers another therapeutic avenue for OSCC treatment. This can be achieved through probiotic interventions, such as using strains that produce beneficial metabolites like indole-3-propionic acid (IPA), which has anti-inflammatory and anti-cancer properties (Zhang et al., 2025; Zhong et al., 2025). By promoting the growth of beneficial microbes or using probiotics that restore the microbial balance, it may be possible to inhibit OSCC progression (Liu et al., 2025). Conversely, antimicrobial therapies targeting bacteria that produce harmful metabolites like H_2_S or KYNA could help reshape the oral microbiome to reduce cancer-promoting factors.

#### 3.2.4 Metabolite supplementation

Supplementing beneficial metabolites directly could offer a supportive therapeutic strategy for OSCC. For example, the local or systemic administration of IPA, a metabolite produced by beneficial microbiota, could be considered as a supplementary treatment. IPA has shown potential in reducing inflammation and promoting immune responses against cancer, making it a promising adjunct to conventional cancer therapies.

This structured approach not only emphasizes the promising role of microbial metabolites as diagnostic biomarkers but also highlights the innovative therapeutic strategies that can be developed around them. By understanding the intricate interplay between microbial metabolites and OSCC progression, we can pave the way for more precise, personalized diagnostic tools and targeted treatments. **Table 2** therefore summarizes the current evidence landscape by stratifying key metabolite axes according to human cohort/metabolomics findings, *in vitro* mechanistic support, and *in vivo* validation, while also outlining major confounders (e.g., smoking, alcohol exposure, HPV status, and periodontal inflammation). Harnessing the power of these microbial byproducts could significantly improve early detection, risk assessment, and treatment outcomes in OSCC, offering new hope for better management and prognosis of this aggressive cancer. Furthermore, as our understanding of the microbiome’s influence on cancer continues to grow, microbial metabolites will likely become an essential part of a multi-faceted approach to OSCC prevention, diagnosis, and therapy.

**Table 2:** Evidence stratification for translational interpretation.

Metabolite/axis	Human evidence	Mechanistic (in vitro) evidence	In vivo evidence	Direction in OSCC	Potential application	Key limitations/confounders
KYNA / kynurenine axis	Reported in saliva/tissue metabolomics; association with OSCC/precancer in some cohorts	Receptor/enzyme signaling described in cancer systems	Limited OSCC-specific in vivo causal tests	Often ↑	Biomarker; enzyme- targeting (IDO1/TDO2); receptor targeting (GPR35)	Strong confounding (inflammation, immune tone); need longitudinal validation
Butyrate (oral SCFA)	Detected in saliva/GCF; varies with periodontal state	HDAC inhibition and immunomodulation shown; dose-dependent effects	Oral-cavity-relevant in vivo models limited	Context-dependent	Mechanistic probe; stratified biomarker panel	Concentration in oral cavity lower than gut; niche- specific exposure
H_2_S / VSCs	Salivary VSCs associated with halitosis/periodontal inflammation; OSCC links emerging	Mitochondrial/redox and DNA damage mechanisms shown in multiple systems	Some animal evidence in inflammation contexts; OSCC-specific limited	Often ↑	Risk marker; microbiome intervention target	Smoking/alcohol and periodontal disease confound; measurement standardization needed
Acetaldehyde	Strong human exposure literature (alcohol + oral microbes); saliva measurable	DNA adduct formation and mutagenesis well supported	Exposure models exist; oral OSCC translation requires careful design	↑ with alcohol	Risk stratification; prevention (reduce exposure)	Alcohol intake dominates; requires careful covariate control
Polyamines	Reported in cancer metabolomics; OSCC- specific patterns variable	Cell proliferation, stress adaptation, immune modulation known	OSCC-specific causal evidence limited	Variable	Biomarker panel component; metabolic targeting concept	Diet and inflammation influence; interindividual variability
Bile acids (salivary)	Observed especially with reflux; OSCC-specific evidence limited	Epithelial stress pathways plausible (FXR/TGR5), not definitive in oral mucosa	Limited	Variable	Context marker (reflux- associated exposure)	Reflux status critical; weak OSCC causality currently

Several critical gaps must be addressed to advance the field. First, establishing conclusive causal inference requires moving beyond cross-sectional human studies, which are confounded by factors like smoking, HPV status, and oral inflammation. Longitudinal cohorts with multi-omics integration are needed to delineate temporal dynamics. Second, the spatial biology of metabolite exposure is poorly understood; determining whether key signals originate from intratumoral bacteria, surface biofilms, or adjacent niches is crucial, as local concentration gradients likely dictate functional outcomes. Third, methodological standardization is imperative, encompassing absolute quantification of metabolites, benchmarking against physiological oral ranges, and linking microbial genomic potential to metabolic output. Finally, a paucity of interventional studies hinders translation. Future work must identify druggable nodes (e.g., microbial enzymes, host receptors) and rigorously evaluate target engagement and safety in relevant preclinical models. Resolving these questions is essential to transform correlative observations into mechanistic biomarkers and actionable therapies for OSCC.

At the same time, some limitations and challenges need attention. Current evidence is constrained by confounding (e.g., smoking, alcohol exposure, HPV status, periodontal inflammation, and oral hygiene), reverse causality (tumor-driven ecological changes), and sampling variability across matrices (tumor-adjacent tissue vs tumor surface, saliva, dental plaque, and gingival crevicular fluid). In addition, metabolite concentrations are highly niche-dependent, and many studies lack absolute quantification and standardized workflows, complicating cross-cohort comparisons and causal attribution.

## 4 Discussion

In conclusion, the intricate interaction between the oral microbiota, its metabolic products, and the host’s cellular environment plays a crucial role in the initiation and progression of OSCC (Wei et al., 2025). The “microbiome-metabolite-host” axis is central to understanding how microbial dysbiosis and the metabolites produced by oral bacteria contribute to the molecular mechanisms of OSCC development (Agrawal et al., 2025). Through this axis, microbial communities are not only passive bystanders but active contributors to cancer biology, influencing key processes such as immune modulation, inflammation, DNA integrity, and cellular metabolism (Liu et al., 2022).

Microbial metabolites, such as butyrate, H_2_S, and kynurenine, have been implicated in modulating host immune programs and inflammatory features of the TME, with supporting evidence derived from a combination of clinical associations and preclinical mechanistic studies (Thakur et al., 2025). For instance, butyrate, while known for its anti-inflammatory and tumor-suppressive effects at higher concentrations, can also enhance tumor cell proliferation at lower levels, depending on the microbial composition and the context within the TME. Similarly, H_2_S, produced by pathogenic bacteria, can induce DNA damage, further exacerbating the carcinogenic process. The kynurenine pathway, mediated by bacteria like *S. mutans*, contributes to immune suppression by promoting Tregs expansion and facilitating an immunosuppressive environment that helps the tumor evade immune surveillance (Gan et al., 2024).

Understanding the complex interactions within this “microbiome-metabolite-host” axis is crucial for uncovering the underlying mechanisms of OSCC and provides significant insight into cancer biology. Importantly, it opens up new avenues for precision diagnostics and therapeutic strategies. As research progresses, one promising approach is the use of metabolomics and metagenomics to identify the specific metabolic signatures associated with OSCC. The integration of these two cutting-edge techniques will allow researchers to deeply analyze the distinct metabolic profiles of OSCC, shedding light on unique cancer-specific biomarkers that reflect microbial imbalances and metabolic disruptions within the oral cavity. These analyses could lead to the development of more sensitive and specific diagnostic tools for early detection and risk prediction in OSCC, enabling more timely interventions.

Metabolomic and metagenomic analyses can reveal not only the specific microbial species involved in OSCC but also their metabolic products, which play pivotal roles in tumor progression (Magrin et al., 2020). Profiling saliva, tissue samples, and other biological fluids for metabolites like elevated kynurenine or altered SCFAs could offer valuable biomarkers for identifying OSCC at its earliest stages, even before the appearance of clinical symptoms (Baima et al., 2025). These biomarkers could also be used to assess the risk of malignant transformation in patients with precancerous lesions, providing a basis for more personalized monitoring and treatment strategies.

Moreover, targeted modulation of microbial metabolic activities represents an emerging and largely preclinical frontier in OSCC prevention and intervention research. By focusing on specific metabolic pathways that are disrupted in the tumor microbiome, researchers can explore new ways to modulate the oral microbiota to prevent or slow the progression of OSCC. For example, experimental strategies targeting enzymes in the kynurenine pathway or limiting hydrogen sulfide–producing bacteria have been proposed to mitigate immunosuppressive and pro-inflammatory conditions that support tumor progression (Abd El-Fattah, 2022; Xu et al., 2023). Restoring microbial balance through probiotics, prebiotics, or other microbiome-modulating approaches has been suggested as a potential strategy, although its efficacy and safety in OSCC remain to be established.

Targeting microbial metabolites directly also offers a promising therapeutic approach. For instance, metabolite supplementation (e.g., indole derivatives such as IPA) has shown immunomodulatory effects in preclinical models, though its translational relevance to OSCC requires further investigation. Additionally, the development of inhibitors for enzymes that produce cancer-promoting metabolites could prevent the accumulation of harmful metabolites and reduce the pro-tumorigenic effects within the oral cavity (Wei et al., 2025).

In the future, a more detailed understanding of the metabolic characteristics of OSCC will be essential in providing novel strategies for the prevention and precision treatment of this aggressive cancer. The combination of metabolomics and metagenomics offers an unparalleled opportunity to uncover cancer-specific metabolic signatures and to develop targeted therapeutic strategies. With continued research in this field, we could see the emergence of microbiome-based treatments that not only target the metabolic pathways that contribute to OSCC but also enhance the effectiveness of existing therapies, providing a holistic approach to cancer treatment that is tailored to the unique microbial and metabolic landscape of each patient.

Despite the growing interest in microbial metabolites as diagnostic biomarkers and therapeutic entry points in OSCC, several important confounding factors must be carefully considered when interpreting metabolomic readouts. Host-related variables, including tobacco smoking, alcohol consumption, human papillomavirus (HPV) status, periodontal inflammation, oral hygiene practices, prior antibiotic or probiotic exposure, and dietary habits, can all substantially influence the composition of the oral microbiome and the abundance of microbial metabolites in saliva, plaque, and tumor-adjacent tissues. These factors may introduce significant variability and complicate causal inference, particularly in cross-sectional studies. To enhance translational robustness, future studies should adopt standardized sampling windows, comprehensive metadata collection, and appropriately matched control cohorts. In addition, integrating metabolomics with metagenomics, transcriptomics, and host clinical covariates, combined with rigorous statistical adjustment, will be essential for disentangling disease-specific metabolic signatures from confounding effects, especially in the context of biomarker discovery and clinical translation.

The “microbiome-metabolite-host” axis represents a critical and underexplored aspect of OSCC research. As we continue to unravel the complex interactions between oral microbes, their metabolites, and the host, it becomes increasingly clear that microbial metabolites are not only biomarkers for diagnosis and prognosis but also potential therapeutic targets. By harnessing the power of metabolomics and metagenomics, we can develop more effective strategies for early detection, risk prediction, and personalized treatment, offering new hope for patients suffering from OSCC.
